# A TGF-loading hydrogel scaffold capable of promoting chondrogenic differentiation for repairing rabbit nasal septum cartilage defect

**DOI:** 10.3389/fbioe.2022.1057904

**Published:** 2022-11-18

**Authors:** Dan Zhang, Ying Su, Peng Sun, Xingzhi Liu, Lin Zhang, Xuwei Ling, Yuhui Fan, Kang Wu, Qin Shi, Jisheng Liu

**Affiliations:** ^1^ Department of Otolaryngology, The First Affiliated Hospital of Soochow University, Soochow University, Suzhou, Jiangsu, China; ^2^ Department of Orthopaedics, The First Affiliated Hospital of Soochow University, Orthopaedic Institute, Soochow University, Suzhou, Jiangsu, China, Suzhou, Jiangsu, China; ^3^ Department of Otolaryngology, The Affiliated Suzhou Hospital of Nanjing Medical University, Suzhou Municipal Hospital, Gusu School, Nanjing Medical University, Suzhou, China

**Keywords:** nasal septum, cartilage, gelatin methacrylate, transforming growth factor-β1, bone mesenchymal stem cell

## Abstract

Hydrogel-based tissue engineering has been widely used to repair cartilage injury. However, whether this approach can be applied to treat nasal septum cartilage defects remains unclear. In this study, three gelatin methacrylate-based scaffolds loaded with transforming growth factor (TGF)-β1 (GelMA-T) were prepared, and their effects on repair of nasal septum cartilage defects were examined. *In vitro*, the GelMA-T scaffolds showed good biocompatibility and promoted the chondrogenic differentiation of bone mesenchymal stem cells. Among three scaffolds, the 10% GelMA-T scaffold promoted chondrogenic differentiation most effectively, which significantly improved the expression of chondrocyte-related genes, including *Col II*, *Sox9*, *and ACAN*. *In vivo*, 10% GelMA-T scaffolds and 10% GelMA-T scaffolds loaded with bone mesenchymal stem cells (BMSCs; 10% GelMA-T/BMSCs) were transplanted into a nasal septum cartilage defect site in a rabbit model. At 4, 12, and 24 weeks after surgery, the nasal septum cartilage defects exhibited more complete repair in rabbits treated with the 10% GelMA-T/BMSC scaffold as demonstrated by hematoxylin & eosin, safranine-O, and toluidine blue staining. We showed that GelMA-T/BMSCs can be applied in physiological and structural repair of defects in nasal septum cartilage, providing a potential strategy for repairing cartilage defects in the clinic.

## Introduction

Nasal septum (NS) perforation is a permanent injury that occurs across both sides of the nasal septum and affects 10% of the population worldwide. This condition is caused by various external factors such as surgery, trauma, inflammation, infection, malignancy, and inhalation (Gold et al., 2017; Kridel and Delaney, 2019) and may lead to pain, bleeding, nasal congestion, scabs, and nasal whistles (Wong and Raghavan, 2010). In patients with NS perforation, scabs that form around the perforation may gradually enlarge and further deteriorate the site of perforation (Watson and Barkdull, 2009), giving rise to mechanical instability and eventual breakdown of the NS structure. Hence, maintaining and restoring the NS structure is crucial in treatment of NS perforation.

Cartilage is an essential structural component of the NS and is prone to impairment after NS perforation. NS cartilage consists mainly of natural cartilage tissue with a few nerves and blood vessels, making it challenging to repair and regenerate this structure (Setton et al., 1999). Natural or synthetic biomaterials for regenerative medicine, such as alginate, chitosan, poly L-lactic acid/poly (glycolic acid), agarose, hyaluronic acid, and proteins (e.g., collagen and gelatin), show potential for use in repairing and regenerating articular cartilage, as well as cartilage associated with NS perforation (Campos et al., 2019). The biomaterial gelatin methacrylate (GelMA) is a light-curing hydrogel with shape-adaptive properties, tunable mechanical properties, biocompatibility, controllable degradation, and cell/drug-laden capability (Xiao et al., 2019; Qiao et al., 2020; Piao et al., 2021; Vanaei et al., 2021; Chen et al., 2022; He et al., 2022), and has been used extensively to repair both soft tissues (cartilage) and hard tissues (bone) (Hu et al., 2020; Qiao et al., 2020; Jiang et al., 2021; Tang et al., 2021; Wang and Wang, 2022). However, pure GelMA does not effectively repair cartilage defects due to its limited capability to induce chondrogenic differentiation. Thus, exogenous cytokines are added to GelMA to enhance its biological function. Transforming growth factor-β1 (TGF-β1) is a multifunctional polypeptide that can promote chondrogenic differentiation of bone mesenchymal stem cells (BMSCs) through transcriptional activation of the Smad signaling pathway (Ju et al., 2022). However, because of its intrinsic instability and rapid enzymatic degradation *in vivo*, TGF-β1 must be delivered at high therapeutic doses that may cause adverse side effects, such as fibrotic disorders. Therefore, TGF-β1 should be loaded into a carrier to improve its efficiency (van Beuningen et al., 1998; Kim et al., 2015). Wu et al. (2020) developed a TGF-β1-loaded biodegradable silk fibroin-gelatin porous scaffold that allows sustained release of TGF-β1 and promotes chondrogenic differentiation of BMSCs *in vitro*. We previously developed a GelMA hydrogel modified with a TGF-β1-affinity peptide that self-recruits endogenous TGF-β1 to promote the chondrogenic differentiation of BMSCs and induce regeneration of articular cartilage (Ju et al., 2022).

The lack of endogenous mesenchymal stem cells (MSCs) at the defect site is another challenge in cartilage repair. Due to the lack of endogenous stem cells in the NS, supplementation with exogenous MSCs has been used in the treatment of cartilage damage. BMSCs can be acquired easily and are ideal seed cells for chondrogenic differentiation and cartilage restoration; these cells play important roles in tissue engineering for cartilage repair (Li and Cui, 2021). For instance, Zhu et al. (2022) developed an injectable and thermally responsive BMSC-loaded hydrogel that prevented cartilage destruction by inducing chondrogenic differentiation of BMSCs *in vivo*. However, numerous studies have demonstrated that chondrogenic differentiation of BMSCs *in vivo* is significantly affected by the surrounding mechanical environment (Guo et al., 2016; Remya and Nair, 2016; Xie et al., 2019). Therefore, designing and optimizing the mechanical properties of the BMSC substrate or carrier is important to promote chondrogenic differentiation.

In the current study, a TGF-β1-loaded GelMA scaffold (GelMA-T) with sustainable release of TGF-β1 was prepared by simply incorporating TGF-β1 into GelMA scaffolds. To optimize the mechanical properties of GelMA for repairing defective NS cartilage, three types of scaffolds (5%, 10%, and 15% GelMA-T) with different mechanical properties were prepared. The adhesion, proliferation, and chondrogenic differentiation of BMSCs cultured on the surface of GelMA-T scaffolds *in vitro* were examined. Furthermore, an animal model of defective NS cartilage was established to assess the regenerative potential of the 10% GelMA-T scaffold and the 10% GelMA-T scaffold loaded with BMSCs (GelMA-T/BMSCs). This tissue-engineered scaffold was found to promote the chondrogenic differentiation of BMSCs and the repair of NS cartilage defects.

## Materials and methods

### Material preparation

GelMA was synthesized as previously described (He et al., 2022). Gelatin (20 g; G1083977, Aladdin Biochemical Technology, Shanghai, China) was dissolved in 200 ml of phosphate-buffered saline (PBS, G101, Vazyme, Nanjing, China) at 60°C. Subsequently, 16 ml methacrylate anhydride (M201529, Aladdin) and 800 ml PBS were added to the gelatin solution. After stirring for 2 h, the reaction solution was collected, placed in a 14–16 kD dialysis bag, and dialyzed. Two weeks later, the GelMA solution was collected and stored at −80°C for 1 d. Finally, the GelMA solution was freeze-dried for 1 week to obtain a powder.

To prepare the GelMA scaffold, the freeze-dried GelMA powder was dissolved in a photoinitiator (2-hydroxy-4′-(2-hydroxyethoxy)-2-methylproplophenone, CAS# 106797-53-9, Tokyo Chemical Industry, Tokyo, Japan) at 65°C for 15 min in the dark. The ratio of GelMA powder weight to photoinitiating solution volume was 0.05, 0.1, and 0.15 g/ml to obtain 5%, 10%, and 15% GelMA precursor solutions, respectively. TGF-β1 (10 ng/ml) was added to the 5%, 10%, and 15% GelMA precursor solutions. Finally, the three precursor solutions were transferred into a mold and allowed to form a gel under 405 nm ultraviolet light to prepare the 5%, 10%, and 15% GelMA-T scaffolds.

### Characterization of molecular structure and microstructure

The chemical composition of the GelMA-T scaffolds was analyzed using attenuated total internal reflectance Fourier transform infrared spectrometry (VERTEX80, Bruker, Billerica, MA, United States) over a wavelength range of 4,000−500 cm^−1^.

The microstructure of the GelMA-T scaffold was characterized using scanning electron microscopy, and porosity was calculated using ImageJ software (NIH, Bethesda, MD, United States). Briefly, the GelMA-T scaffolds were freeze-dried and brittle-fractured to expose the cross-sectional structure. The freeze-dried hydrogel was sputtered at 18 mA for 60 s using an ion-sputtered coater (SC7620, Quorum Technologies, East Sussex, United Kingdom) and photographed using a scanning electron microscope (Quanta 250, FEI, Hillsboro, OR, United States). Finally, OriginPro2021 software (Northampton, MA, United States) was used to analyze the size distribution of pores.

### Rheological test

The rheological properties of the GelMA-T scaffolds (1 mm in thickness and 20 mm in diameter) were determined using a Discovery Hybrid 2 (DHR-2) Rheometer (TA Instruments, New Castle, DE, United States) equipped with a temperature control unit in oscillating mode using a plate-plate configuration (plate diameter of 20 mm, with a 1 mm gap between the upper and lower plates). Frequency sweep tests were performed from 0.1 to 10 Hz at 37°C and 1% strain.

### Mechanical test

The compressive mechanical properties of the GelMA-T scaffolds were tested using a universal mechanical testing machine (HY-0580; Shanghai Hengyi Co., Ltd., Shanghai, China) at a compression speed of 10 mm/min. The slope of the strain in the first 10% of the stress–strain curve was defined as the compressive elastic modulus of the sample, which reflected its stiffness.

### Release test

The release of TGF-β1 from the GelMA-T scaffold was evaluated as previously reported (Zhu et al., 2018). As a model protein, bovine serum albumin (BSA) was incorporated into the scaffold in place of TGF-β1 to evaluate the release profile. Briefly, BSA (A1933, Sigma, St. Louis, MO, United States) was added to 5%, 10%, and 15% GelMA scaffolds. The BSA-loaded GelMA scaffolds were placed in dialysis bags and immersed in 5 ml PBS for incubation at 37°C. The extract (20 μl) of the scaffold was collected at predetermined time points (1, 2, 3, 4, 5, 6, and 7 days). Finally, the protein concentration of the collected solution was measured using a BCA kit (P0010; Beyotime, Shanghai, China), and the curve representing the cumulative release of BSA from the GelMA scaffold was plotted to predict the capacity for release of TGF-β1 from the GelMA scaffold.

### *In vitro* degradation and swelling test

The degradation properties of the scaffolds were tested, as previously reported (Rehman et al., 2022), by comparing the dry weights of the scaffolds before and after soaking in deionized water. Briefly, all samples were placed in an oven at 60°C and dried to a constant weight, and the initial weight (W_0_) was measured. The samples were immersed in deionized water (ratio of sample weight to water volume was 0.1 g/ml) for 7, 14, and 28 days. The dry weights of the scaffolds (W_t_) at 7, 14, and 28 days were measured, and the degradation rate was calculated according to the following formula:

$$Mass\;loss\;rate\;\left(\%\right)=\frac{W_{0}-W_{t}}{W_{0}}\times 100\%.$$

The swelling ratio was evaluated by comparing the initial dry weight (W_0_) of scaffolds and wet weight (W_t_) of scaffolds after immersion in the deionized water for 24 h. The swelling ratio was calculated according to the following formula:

$$Swelling\;ratio\;\left(\%\right)=\frac{W_{t}-W_{0}}{W_{0}}\times 100\%.$$

### Isolation, culture, and identification of rabbit-derived BMSCs

Following the previously reported method (Cheng et al., 2014), BMSCs were isolated from 2-month-old New Zealand white rabbits. Briefly, 5 ml bone marrow was extracted from the tibia of the rabbits under aseptic conditions and then centrifuged at 800 rpm for 5 min to remove the fat granules and supernatant. The bone marrow was carefully added to tubes containing 4 ml of pre-set lymphocyte separating medium (density: 1.073 kg/L, Sigma) and then subjected to gradient centrifugation at 2,000 rpm for 30 min. The mononuclear cells were isolated, washed twice with Hanks buffer, and seeded at 5 × 10^4^ cm^−2^ into alpha-modified minimum essential medium (α-MEM, HyClone, Logan, UT, United States) for incubation at 37°C with 5% CO_2_. When the primary cells became adherent and reached 80% confluence, they were detached using 0.25% trypsin (C100C1; New Cell & Molecular Biotech Co., Ltd.) for further passaging. Cells from the third passage were prepared as cell suspensions at a concentration of 10^6^ cells/ml. The cells were identified as rabbit-derived BMSCs using the cell markers CD29 (11-0291-82, Thermo Fisher Scientific, Waltham, MA, United States) and CD90 (MA1-80650, Thermo Fisher Scientific) and confirmed to not express CD34 (MA1-22646, Thermo Fisher Scientific) or CD45 (MA5-28392, Thermo Fisher Scientific) using the Attune™ NxT Acoustic Focusing Cytometer (AFC2, Thermo Fisher Scientific).

### *In vitro* cytocompatibility test

The cytocompatibility of the GelMA-T scaffolds was assayed by culturing BMSCs with the scaffold extract or on the scaffold surface. Briefly, the scaffolds (7 mm in diameter and 3.5 mm in height) were sterilized using UV light for 10 min and then transferred into medium at a 0.1 g/ml ratio of sample to α-MEM and incubated at 37°C for 24 h. The extracts were collected, filtered through a 0.22-μm membrane (Millex-GP; Millipore, Billerica, MA, United States) for further sterilization, and supplemented with 10% (v/v) fetal bovine serum to prepare complete extraction medium. Additionally, cell culture medium composed of α-MEM supplemented with 10% (v/v) fetal bovine serum was cultured as a control. After washing with PBS and trypsinization with 0.25% trypsin-EDTA, BMSCs were seeded into 96-well plates at a density of 5,000 cells/well in cell culture medium. After incubation for 24 h, the medium was replaced with 100 μl scaffold extract. After incubation for 1, 3, and 7 days, cell viability was evaluated using a Cell Counting Kit-8 kit (Dojindo Molecular Technologies, Kumamoto, Japan) by measuring the optical density (OD) values at 450 nm with a microplate reader (PowerWave X, BioTek Instruments, Winooski, VT, United States).

To evaluate the adhesion ability of BMSCs cultured on the scaffold surface, 5%, 10%, and 15% GelMA-T scaffolds were placed in a 24-well plate and sterilized with UV light. BMSCs were seeded onto the surface of the scaffolds at a density of 40,000 cells/well. At 1, 3, and 7 days, live/dead staining and cytoskeleton staining were performed on the BMSCs cultured on the scaffold surface. BMSCs cultured in 24-well plates without scaffolds were used as controls.

For live/dead staining, the BMSCs were stained using a Calcein-AM/PI Double Staining Kit (Dojindo) and imaged under a fluorescence microscope (NR3831001630, Carl Zeiss, Oberkochen, Germany). For cytoskeleton staining, the BMSCs were washed with PBS, fixed with 4% formaldehyde solution (P395744, Aladdin) at 4°C for 25 min, and permeabilized with 0.5% Triton X-100 at room temperature for 10 min. The BMSCs were then incubated with rhodamine-phalloidin (YP0063, YuHeng, China) at room temperature for 20 min. The nuclei were stained with 4′,6-diamino-2-phenyl indole (DAPI; R20274, YuanYe Bio-Technology, Shanghai, China) at room temperature for 15 min. After staining, the BMSCs were washed three times with PBS and imaged using fluorescence microscopy.

### *In vitro* chondrogenic differentiation test

To evaluate whether the scaffolds could promote chondrogenic differentiation, BMSCs at a density of 5,000 cells/well were cultured on the GelMA and GelMA-T scaffolds in α-MEM. As controls, BMSCs were cultured in a plate containing α-MEM with or without addition of TGF-β1 for comparison with the GelMA-T and GelMA scaffolds, respectively. All culture media was changed every 5 days. Chondrogenic differentiation was assessed using immunofluorescence staining and quantitative reverse-transcription polymerase chain reaction (qRT-PCR). After culturing the cells for 21 days, the BMSCs were subjected to immunofluorescence staining and qRT-PCR.

For immunofluorescence staining, the BMSCs were washed with PBS, fixed with 4% paraformaldehyde at 4°C for 30 min, and permeabilized with 0.1% Triton X-100 at room temperature for 15 min. The cells were blocked with 3% BSA at room temperature for 30 min, incubated with primary antibodies against SRY-box transcription factor 9 (SOX9; ab185966, Abcam, Cambridge, United Kingdom) at 4°C overnight, and then incubated with fluorescein isothiocyanate-conjugated goat anti-mouse IgG secondary antibodies (ab288251, Abcam) at 37°C for 1 h. The cell nuclei were stained with DAPI (C1005, Beyotime) for 15 min. After staining, the cells were washed three times with PBS and imaged using fluorescence microscopy (Olympus IX71, Tokyo, Japan).

For qRT-PCR, we measured the expression of the chondrogenic-related genes collagen type II (*Col II*), aggrecan (*ACAN*), and *Sox9*. Briefly, total RNA was extracted using an extraction kit (Cell Total RNA Isolation Kit V2; Vazyme) according to the manufacturer’s instructions. The concentration and purity of the isolated RNA were measured using a spectrophotometer (FLX800T; BioTek). Total RNA was converted into cDNA using a reverse transcription system (Code No. FSQ-201, Toyobo Co., Ltd., Osaka, Japan). The synthesized cDNA was used for qRT-PCR using a real-time PCR detection system (Bio-Rad, Hercules, CA, United States), and gene expression was normalized to that of glyceraldehyde-3-phosphate dehydrogenase and calculated using the 2^−ΔΔCT^ method. In addition, the gene expression levels of cells cultured in regular medium were set to one-fold. All primer sequences are shown in the Supplementary Table S1.

### Animal experiments

Use of animals was approved by the Laboratory Animal Care and Use Committee of Soochow University. Forty-eight (*n* = 4 per group) adult male New Zealand white rabbits (2.5–3.0 kg in weight; Experimental Animal Center of Soochow University, Suzhou, China) were randomly divided into four groups: sham, control, 10% GelMA-T, and 10% GelMA-T/BMSCs. To prepare the 10% GelMA-T/BMSC scaffold, the 10% GelMA-T scaffold was immersed in α-MEM medium and then cultured in an incubator for 12 h. The second passage of the BMSC suspension was pipetted onto the surface of the 10% GelMA-T scaffold in a 24-well plate at approximately 1 × 10^5^ cells per well. After culturing the cells in an incubator for 3 days, 10% GelMA-T/BMSCs scaffolds were obtained and prepared for implantation *in vivo*. Before surgery, the rabbits were weighed and then administered general anesthesia *via* injection with 2% pentobarbital sodium (P11011, Merck, Kenilworth, NJ, United States) at 2 ml/kg in the ear vein using a 5-ml syringe connected to an intravenous infusion needle. Next, 2% lidocaine hydrochloride (612-079-4, Shanghai Zhaohui Pharmaceutical Co., Ltd., Shanghai, China) was used for local anesthetization and administered using a 1-ml syringe at 0.4 ml/kg. To separate the skin and bone, an incision 3 cm in length was established in the center of the nose. A defect 0.5 cm in width and 2 cm in length was created on the nasal bone in the center of the incision by abrasive drilling to expose the nasal cartilage. A half-round defect (diameter, 0.5 cm) was made on the nasal cartilage. For the experimental groups, the half-round defects were filled with 10% GelMA-T and 10% GelMA-T/BMSCs. The incision was sutured, and penicillin was injected intramuscularly for 3 days. The animals were sacrificed at 4, 12, and 24 weeks post-surgery by excessive anesthesia with 2% pentobarbital sodium (4 ml/kg).

### Histological staining

After fixation in 10% neutral formalin for 48 h, the NS cartilage specimens were dehydrated using an alcohol gradient. The specimen was embedded in optimal cutting temperature compound (14020108926 Leica, Wetzlar, Germany) and serially sliced at a thickness of 6 mm using a frozen microtome (CM 1950, Leica). The sections were stained with hematoxylin and eosin, safranine-O, and toluidine blue and visualized using bright-field microscopy (Zeiss Axiovert 200M).

### Statistical analysis

Statistical analysis was performed using GraphPad Prism software (version 8.0, GraphPad, Inc., La Jolla, CA, United States). All experiments were replicated at least three times. The data are expressed as the mean ± standard deviation. One-way analysis of variance with Tukey’s *post hoc* test was used for multiple comparisons. The results were considered statistically significant at *p* < 0.05 or *p* < 0.01.

## Results and discussion

### Material characterization

**FIGURE 1:**
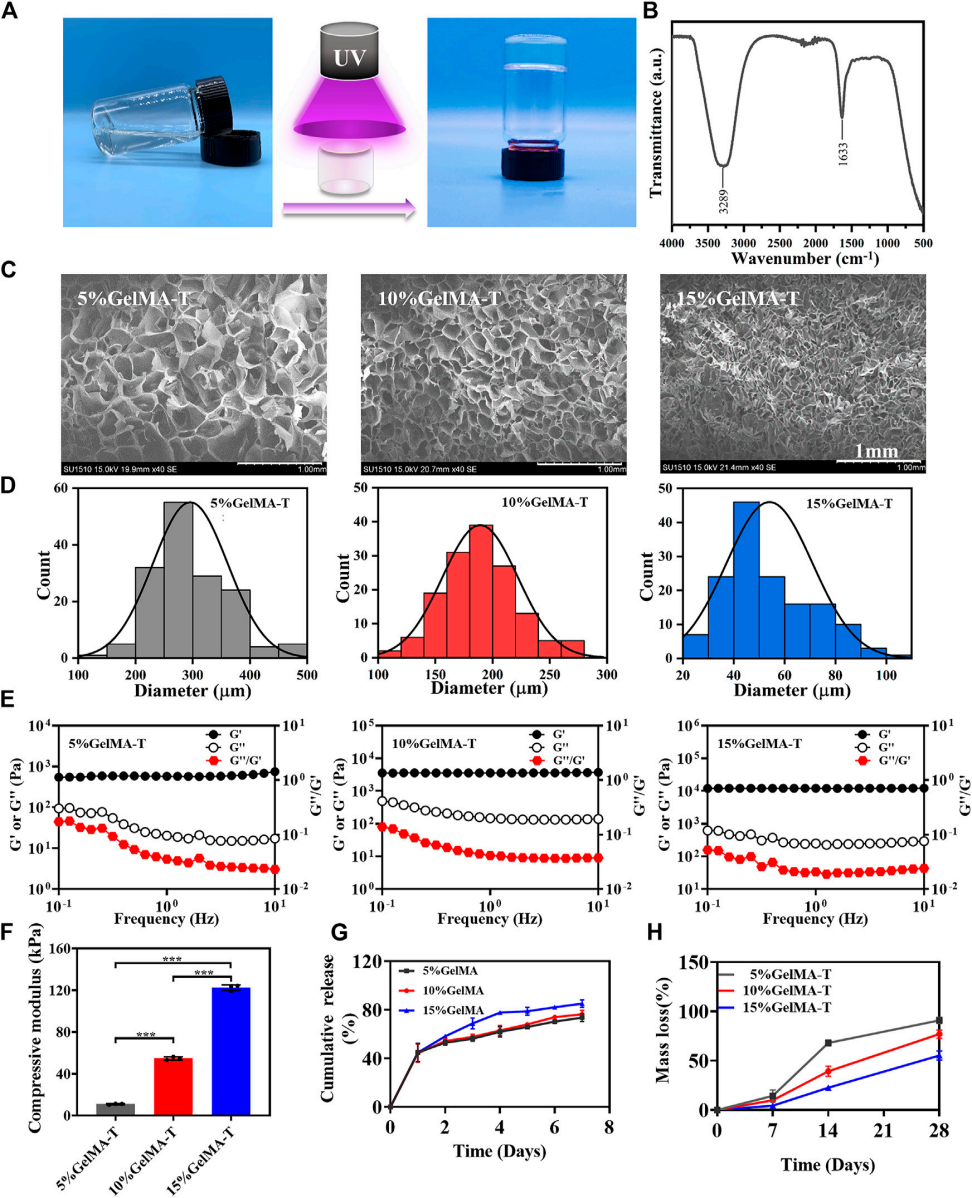
Physicochemical properties of GelMA-T scaffolds. **(A)** GelMA-T scaffold transformed from liquid to solid under UV irradiation. **(B)** Fourier transform infrared spectrum of 10% GelMA-T scaffold. **(C)** Scanning electron microscopy results of three GelMA-T scaffolds, scale bar: 1 mm. **(D)** Pore diameter distribution of three GelMA-T scaffolds. **(E)** Storage and loss modulus of three GelMA-T scaffolds. **(F)** Compressive modulus of three GelMA-T scaffolds. All data are expressed as the mean ± standard deviation (*n* = 3, *p < 0.05, **p < 0.01). **(G)** Cumulative release of BSA from scaffolds. **(H)** Degradable properties of GelMA-T scaffolds at 7, 14, and 28 days. Abbreviations: BSA, bovine serum albumin; GelMA-T, gelatin methacrylate-based scaffolds loaded with transforming growth factor-β1.

To obtain a scaffold with suitable porosity, mechanical properties, and degradation properties for the adhesion, proliferation, and chondrogenic differentiation of BMSCs, GelMA-T scaffolds were prepared in three solid-to-liquid ratios (0.05, 0.1, and 0.15 g/ml). As shown in Figure 1A, GelMA-T is a photo-crosslinking hydrogel that transforms from a liquid to a solid under UV irradiation. The Fourier transform infrared spectroscopy results showed that the GelMA-T scaffolds had two typical absorption peaks. The absorption peak at 3,289 cm^−1^ corresponded to the stretching vibration of the N-H peptide bond, whereas the absorption peak at 1,663 cm^−1^ was the stretching vibration of the C=O double bond (Figure 1B), which is consistent with previously reported results for GelMA (Weng et al., 2020; Da Silva et al., 2021).

Scanning electron microscopy images of the three GelMA-T scaffolds revealed a foam-like porous structure (Figure 1C) that was conducive to cell adhesion and proliferation (Sandri et al., 2015), and the porosity of GelMA-T improved with increasing concentrations. In addition, the pore sizes of 5%, 10%, and 15% GelMA-T scaffolds ranged from 200–500 μm, 100–300 μm, and 20–100 μm, with main distributions of approximately 300, 150, and 50 μm, respectively (Figure 1D). Scaffolds with pore size of 100–200 μm promote cartilage regeneration (Di Luca et al., 2016). Chondrocyte diameter is approximately 60–200 μm (Ng et al., 2003); thus, GelMA-T scaffolds may support the loading and ingrowth of chondrocytes.

The rheological test results showed that the storage modulus (G′) of the 5%, 10%, and 15% GelMA-T scaffolds at 1 Hz were 574.0, 3564.6, and 12,030.3 Pa, respectively; the loss modulus (G′) was 20.4, 147.2, and 248.8 Pa, respectively; and the loss factor (G′′/G′) was 0.04, 0.04, and 0.02, respectively (Figure 1E). The G′ was higher than the G″ for the three scaffolds, indicating that the scaffolds had viscoelastic-solid properties, enabling them to provide mechanical support for the defective NS (de Gabory et al., 2010). The compressive elastic modulus values of the 5%, 10%, and 15% GelMA-T scaffolds were 11.0 ± 0.6, 54.7 ± 1.6, and 122.6 ± 2.7 kPa, respectively (Figure 1F and Supplementary Figure S1), suggesting that the stiffness (elastic modulus) of the scaffold improved with increasing GelMA concentrations.

The result of the release test (Figure 1G) showed that ∼44% of total BSA was released from all three scaffolds with a high release rate on the first day. After release for 7 days, the cumulative amount of BSA slowly increased and gradually reached release plateaus (∼73%, ∼76%, and ∼85% for the 5%, 10%, and 15% scaffolds, respectively). The amount of the released BSA gradually improved with increasing GelMA concentrations, possibly because the higher osmotic pressure of GelMA at high concentrations enhanced the diffusion capability of BSA into the medium. The results suggest that increasing the GelMA concentration prolonged the release period and amount of cargo in the scaffolds, which may provide more sustained stimulation for cell growth and differentiation (Zhu et al., 2018).

The degradation of implanted scaffolds is an important property in cartilage regeneration (Lai et al., 2021). Analysis of the degradable properties of the three GelMA-T scaffolds (Figure 1H) suggested that the mass loss rates of the 5%, 10%, and 15% GelMA-T scaffolds were 14.4%, 10.3%, and 4.4%, respectively, at day 7. After immersion in deionized water for 14 days, the mass loss rates of the 5%, 10%, and 15% GelMA-T scaffolds improved to 68.1%, 39.3%, and 22.7%, respectively. On day 28, the mass loss rates of the 5%, 10%, and 15% GelMA-T scaffolds reached 91.0%, 77.1%, and 55.3%, respectively. The three GelMA-T scaffolds exhibited gradually increasing mass loss when immersed in deionized water. The mass loss rate of the GelMA-T scaffold was negatively related to the GelMA concentration, which was attributed to the strong covalent interaction between the polymer chains in the scaffold. The controllable mass loss rate was advantageous for selecting the scaffold whose degradation rate matched the regeneration rate of NS cartilage. In addition, the swelling ratios of the three scaffolds were 176.8%, 140.4%, and 112.3%, respectively (Supplementary Figure S2), demonstrating that the GelMA-T scaffolds could absorb blood and exudate at the implant site.

### *In vitro* cytocompatibility and cellular adhesion behavior

**FIGURE 2:**
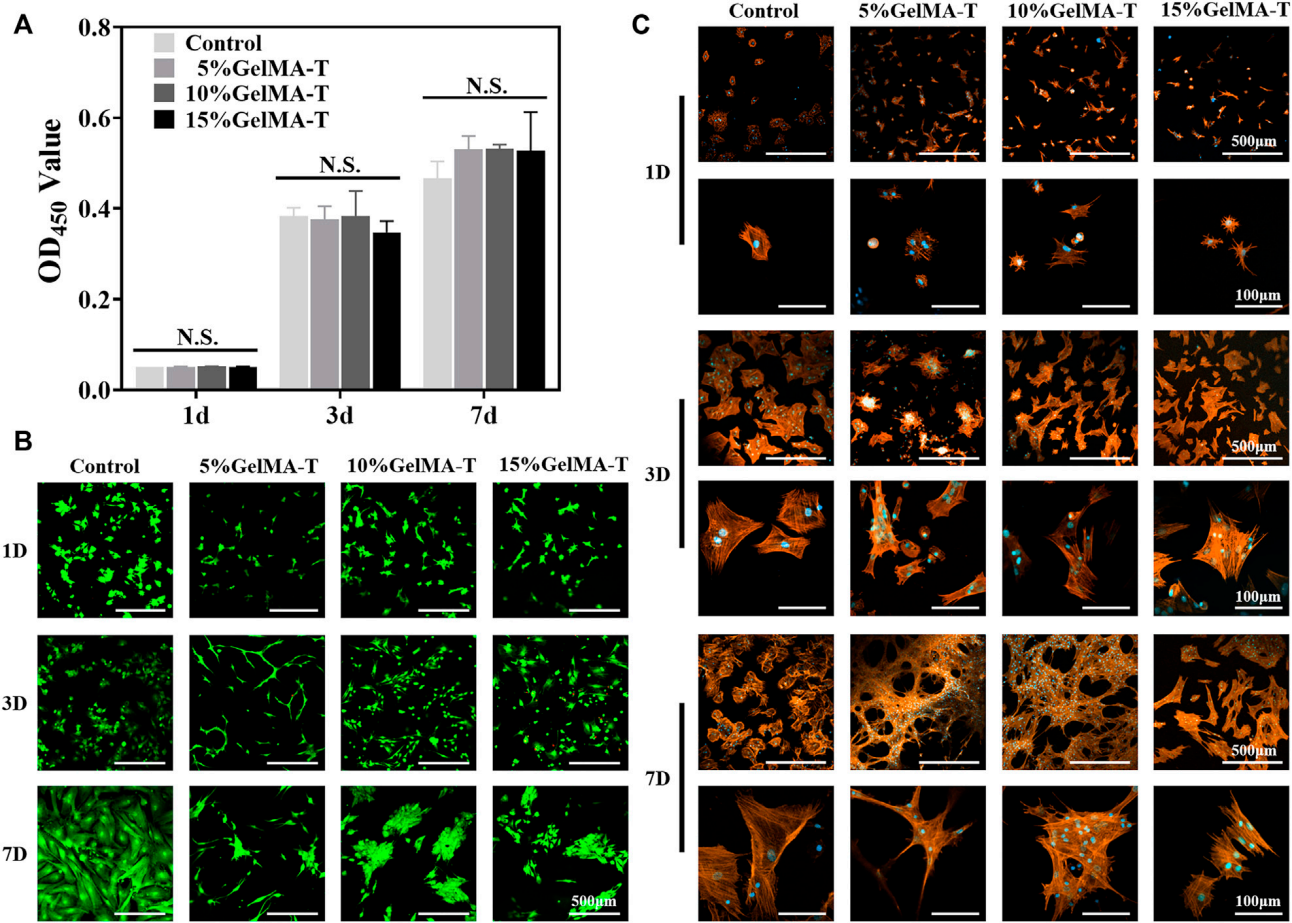
Biocompatibility of GelMA-T scaffold. **(A)** BMSCs cultured for 1, 3, and 7 days were detected using a CCK-8 assay. All data are expressed as the mean ± standard deviation (*n* = 3, *p < 0.05, **p < 0.01, NS, not significant). **(B)** Live (green fluorescence)/dead (red fluorescence) staining of BMSCs cultured for 1, 3, or 7 d. Scale bar: 500 μm. **(C)** Morphology and distribution of BMSCs cultured on GelMA-T scaffolds with different concentrations at days 1, 3, and 7. Scale bar: 500 and 100 μm. Abbreviations: BMSC, bone mesenchymal stem cell; GelMA-T, gelatin methacrylate-based scaffolds loaded with transforming growth factor-β1; CCK-8, Cell Counting Kit-8.

Flow cytometry was performed to quantify BMSC percentage. As shown in Supplementary Figure S3, the ratios of CD90- and CD29-positive cells were both 99.9%, whereas the ratios of CD45- and CD34-positive cells were 0.19% and 0.11%, respectively, demonstrating that the extracted cells were BMSCs (Cheng et al., 2014). The *in vitro* biocompatibility of GelMA-T scaffolds was evaluated as previously reported (Wu et al., 2023). None of the three GelMA-T scaffolds strongly affected the proliferation of BMSCs after 1, 3, and 7 days compared with using α-MEM alone (Figure 2A). Live/dead staining of BMSCs cultured on the surface of the three GelMA-T scaffolds showed that the scaffolds supported the survival and proliferation of BMSCs after culture for 1, 3, and 7 days (Figure 2B). However, compared to the control, BMSCs cultured on the surface of the 10% and 15% GelMA-T scaffolds showed a more round and aggregated morphology, which was commonly observed on a previously reported gel substrate and may be associated with higher expression of chondrogenic markers (Zhong et al., 2013; Qin et al., 2020; Walker et al., 2021). In addition, BMSCs cultured on the surface of 5% GelMA-T scaffolds exhibited a more slender morphology, which may be related to the low stiffness of the scaffolds (Oh et al., 2016).

To determine the capability of the scaffolds to promote adhesion of BMSCs, F-actin in BMSCs cultured on the surface of the GelMA-T scaffolds was assessed via staining at 1, 3, and 7 days. Interestingly, the cell morphology was inconsistent between different groups (Figure 2C). For BMSCs cultured on the surface of 15% GelMA-T scaffolds, the cell spreading pattern was similar to that in the control from 1 to 7 days. In contrast, BMSCs cultured on the surface of 5% and 10% GelMA-T scaffolds showed greater extension of pseudopodium and a larger spreading area after 7 days. These results suggest that the 5% and 10% GelMA-T scaffolds promote the adhesion of BMSCs, which is important in tissue engineering applications.

### Capacity of scaffolds to promote chondrogenic differentiation *in vitro*

**FIGURE 3:**
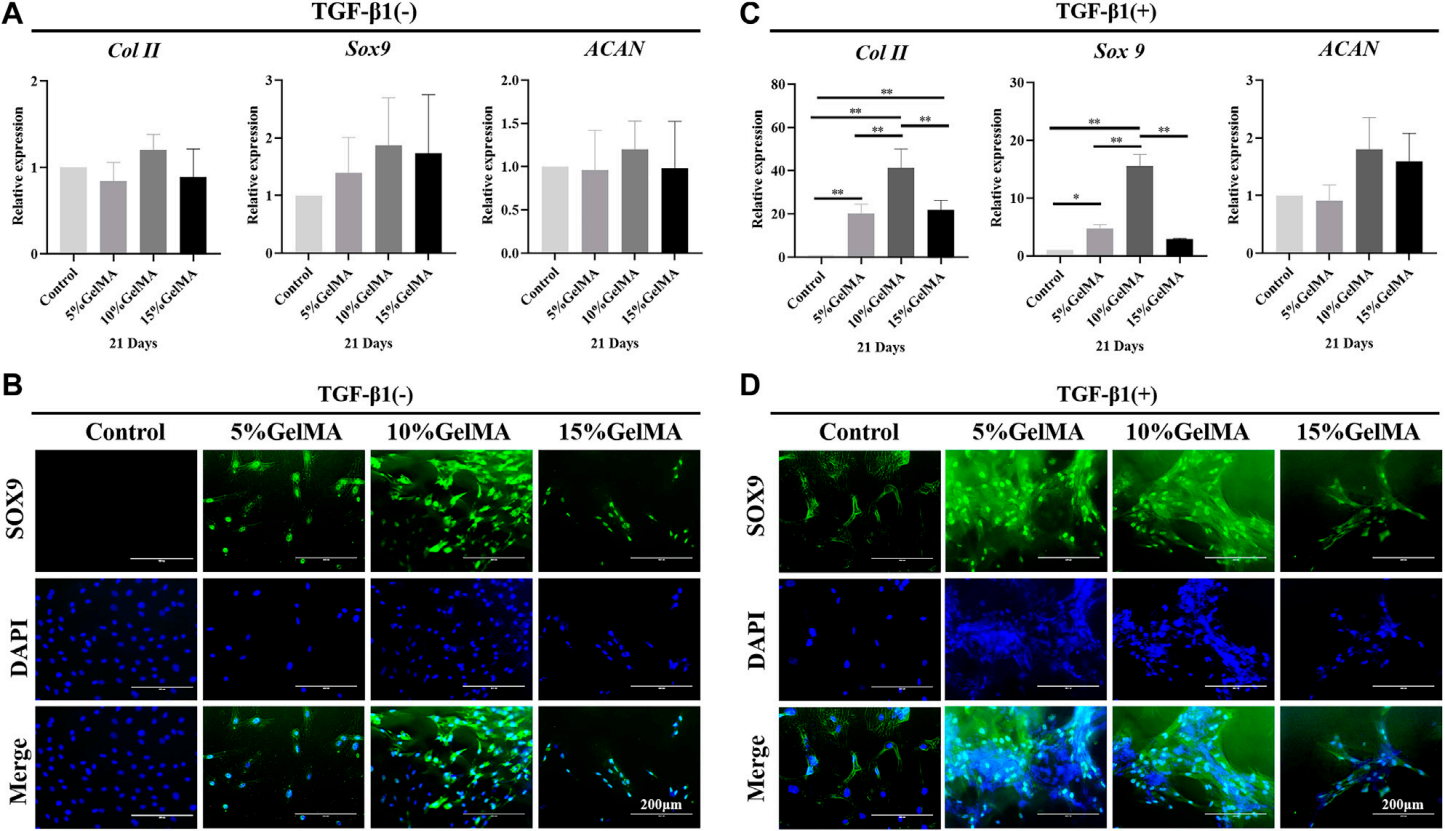
Chondrogenic differentiation-promoting effect of GelMA and GelMA-T scaffolds. **(A)** qRT-PCR analysis of chondrogenic gene expression in BMSCs cultured on the surface of GelMA scaffolds for 21 days. All data are expressed as the mean ± standard deviation (*n* = 3, *p < 0.05, **p < 0.01). **(B)** Immunofluorescence staining of the chondrogenic marker *SOX9* (* SOX9*: green, DAPI: blue) in BMSCs cultured on the surface of GelMA scaffolds for 21 d. **(C)** qRT-PCR analysis of the chondrogenic gene expression of BMSCs cultured on the surface of GelMA scaffolds and supplemented with TGF-β1 for 21 d. All data are expressed as the mean ± standard deviation (*n* = 3, *p < 0.05, **p < 0.01). **(D)** Immunofluorescence staining of the chondrogenic marker *SOX9* (* SOX9*: green, DAPI: blue) in BMSCs cultured on the surface of GelMA scaffolds and supplemented with TGF-β1 for 21 d. Abbreviations: BMSC, bone mesenchymal stem cell; GelMA-T, gelatin methacrylate-based scaffolds loaded with transforming growth factor-β1; qRT-PCR, quantitative reverse-transcription polymerase chain reaction; TGF-β1, transforming growth factor β1.

The expression of cartilage-related genes in BMSCs cultured on the surface of the 5%, 10%, and 15% GelMA scaffolds, including *Col II*, *ACAN*, and *Sox9*, did not differ significantly from that in the control. However, 10% GelMA scaffolds exhibited slightly upregulated expression of the three chondrogenic genes, particularly *Sox9*, which was approximately 1.9-fold higher than that in the control (Figure 3A). The immunofluorescence images suggested that BMSCs cultured with 10% GelMA scaffolds expressed higher levels of SOX9 protein than those in the control cultures (Figure 3B). These results support that 10% GelMA scaffolds can improve both *Sox9* gene and protein expression in BMSCs. *Sox9* controls chondrocyte differentiation by directly activating the expression of chondrogenic genes such as *Col II*, thereby playing an important role in converting BMSCs into chondrocytes (Wu et al., 2015). This likely explains the higher expression of *Col II* in the 10% GelMA scaffold compared to that in the control (Figure 3A). The upregulated expression of three chondrogenic genes in the 10% GelMA scaffolds may have been related to its elastic modulus (54.7 ± 1.6 kPa). A previous study showed that culture substrates with different levels of stiffness induced BMSCs to differentiate into different cell types, with a stiffness of ∼20 kPa for nerve cells, ∼80 kPa for chondrocytes, and ∼190 kPa for osteoblasts (Oh et al., 2016).

After adding TGF-β1, the three GelMA scaffolds showed a significantly enhanced ability to promote the chondrogenic differentiation of BMSCs. As shown in Figure 3C, although *ACAN* expression did not significantly differ between the three scaffolds compared to control, *Col II* expression in BMSCs on the 5%, 10%, and 15% GelMA scaffolds was ∼20.3-, ∼41.3-, and ∼21.7-fold higher than that in the control. *Sox9* expression in BMSCs on the three scaffolds was ∼4.7-, ∼15.6-, and ∼3.0-fold higher than that in the control. Furthermore, the immunofluorescence images suggested that BMSCs in the 5% and 10% GelMA scaffolds expressed significantly higher levels of SOX9 protein compared to SOX9 expression in the other groups (Figure 3D). The enhanced ability to promote chondrogenic differentiation may be related to the effects of TGF-β1, which promotes the chondrogenesis of BMSCs (Futrega et al., 2021).

### Effects of GelMA-T scaffolds on repairing NS cartilage defects *in vivo*

A 5-mm-diameter defect was created on the NS cartilage of rabbits, and the half-round 10% GelMA-T scaffolds or 10% GelMA-T/BMSC scaffolds (5 mm in diameter and 1 mm in thickness) were implanted to fill the defect site. Owing to the swelling capability, the 10% GelMA-T/BMSCs scaffold rapidly swelled after absorbing blood from the nasal bone marrow (Supplementary Figure S4), indicating that some chondrocytes could migrate into the pore of the scaffold due to the swelling effect. Sham, blank control, and 10% GelMA-T scaffolds were used for comparison. No obvious surgical complications were observed in any of the groups throughout the experiments.

**FIGURE 4:**
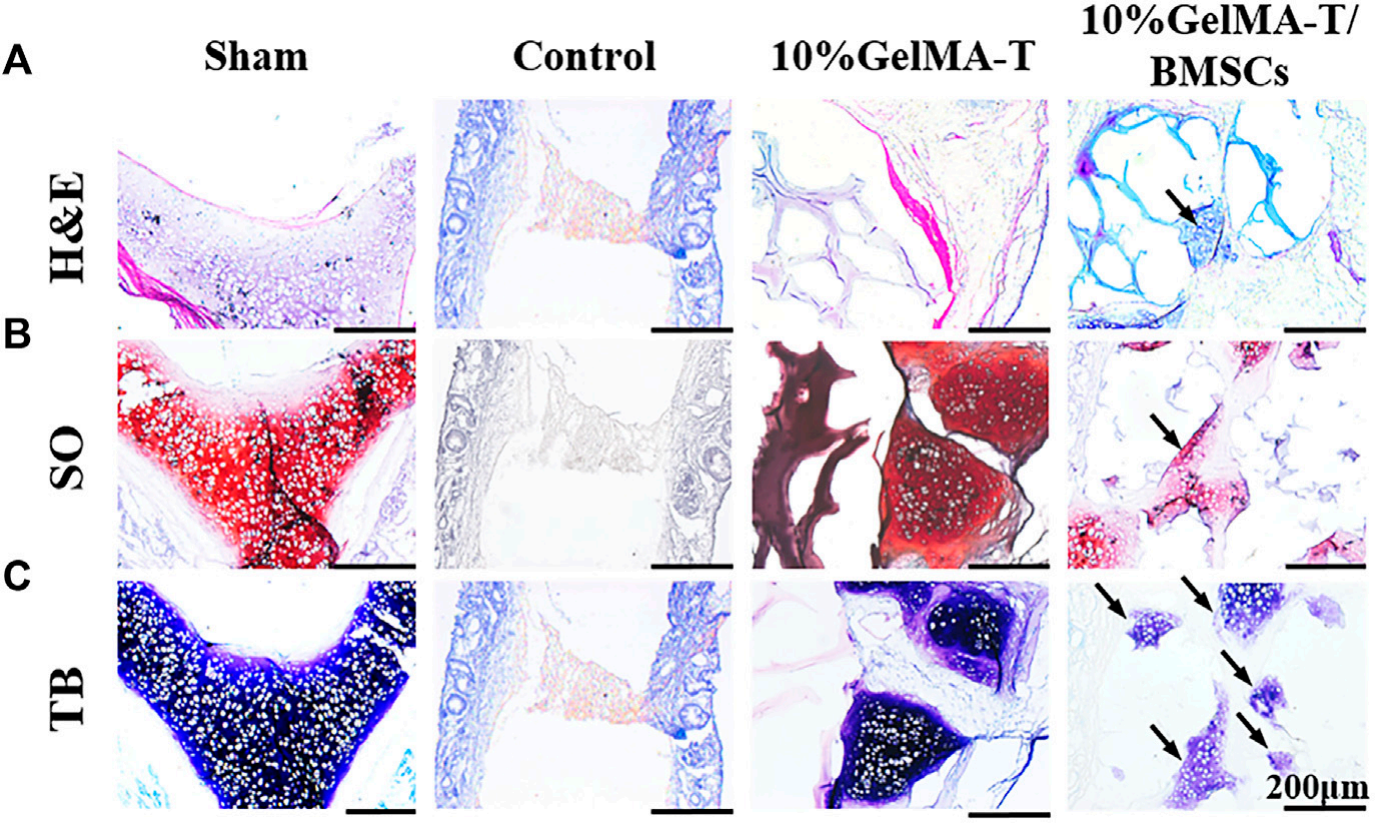
Representative image of rabbit NS cartilage repaired by 10% GelMA-T and 10% GelMA-T/BMSCs scaffolds at 4 w. **(A)** H&E, **(B)** SO, and **(C)** TB staining of NS cartilage in different groups. Scale bar: 200 μm. Abbreviations: GelMA-T, gelatin methacrylate-based scaffolds loaded with transforming growth factor-β1; GelMA-T/BMSCs, GelMA-T scaffolds loaded with bone mesenchymal cells; H&E, hematoxylin and eosin; NS, nasal septum; SO, safranine-O; TB, toluidine blue.

A coronal section of the NS cartilage was assessed using histological staining (Figure 4) to evaluate the gradual regeneration of the NS cartilage during repair. As shown in Figure 4A, at week 4 after surgery, the sham group showed an intact NS cartilage structure and dense expression of proteoglycans (red) and chondrogenic matrix (blue). However, the self-healing capacity of the control was insufficient to reconstruct the defect in the NS cartilage, which was filled with fibrous and connective tissue that likely permeated from the mucous on both sides. Minimal expression of proteoglycans (red) and chondrogenic matrix (blue) was observed. For NS cartilage treated with the 10% GelMA-T scaffold, residual scaffold structure was still detected because of incomplete degradation *in vitro*. Compared to the 10% GelMA-T scaffold, the tissue-engineered 10% GelMA-T/BMSCs scaffold showed a similar residual scaffold structure in the defect site, whereas some laden BMSCs (marked by arrow) were found in the porous structure of the 10% GelMA-T/BMSCs scaffold (Figure 4A). These laden BMSCs promoted the formation of proteoglycans (red) and chondrogenic matrix (blue) at the defect site (Figures 4B,C). NS cartilage treated with the 10% GelMA-T/BMSCs scaffold showed very little immune cell infiltration and no signs of fibrotic tissue at the defect site. These results indicate that the tissue-engineered implant of BMSCs can supply exogenous seed cells for repair of defective NS cartilage, and that TGF-β1 released from GelMA-T promotes the synthesis of chondrocyte-like matrix in BMSCs (Ju et al., 2022). Thus, combined use of tissue-engineered BMSCs and TGF-β1 is a potential repair mechanism for NS cartilage defects.

**FIGURE 5:**
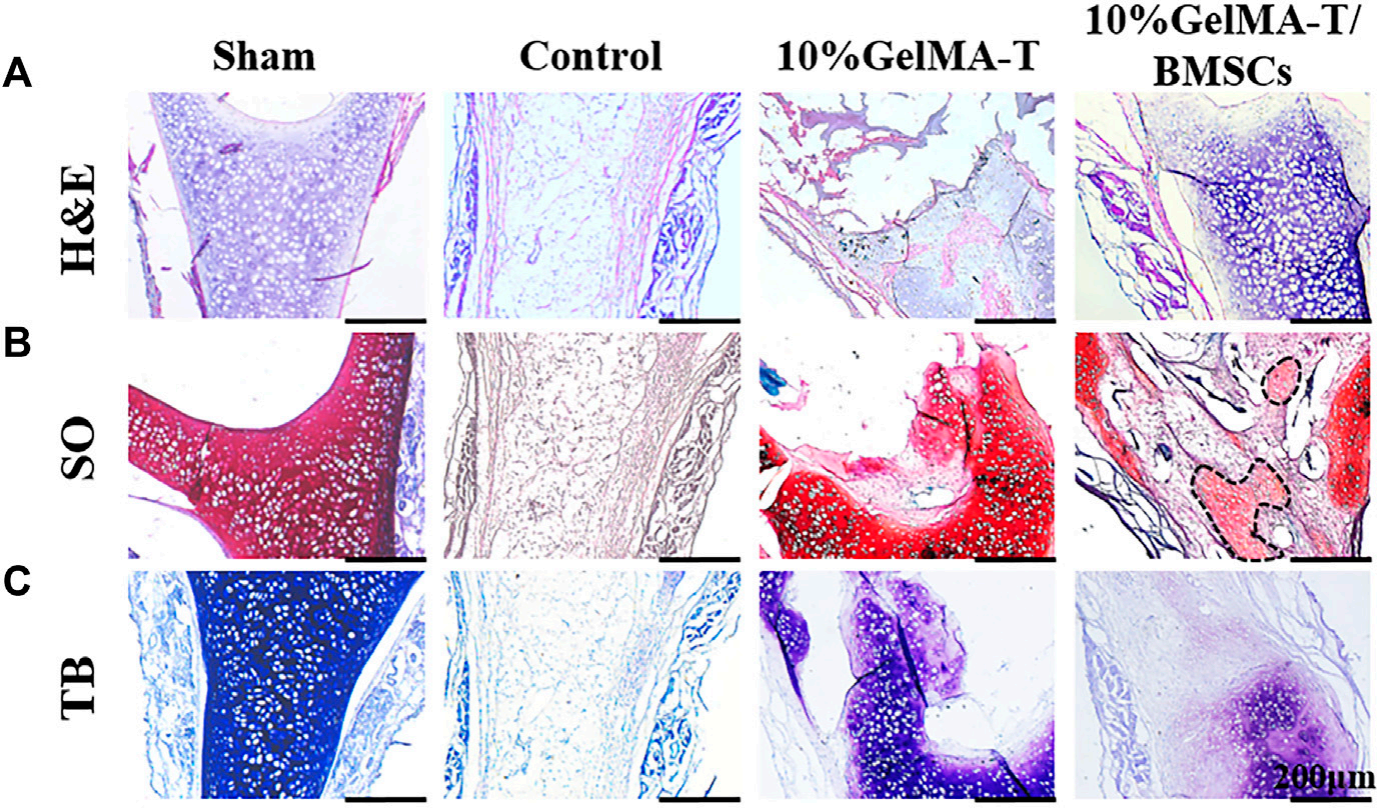
Representative image of rabbit NS cartilage repaired using 10% GelMA-T and 10% GelMA-T/BMSC scaffolds at 12 weeks. **(A)** H&E, **(B)** SO, and **(C)** TB staining of NS cartilage in different groups. Scale bar: 200 μm. Abbreviations: GelMA-T, gelatin methacrylate-based scaffolds loaded with transforming growth factor-β1; GelMA-T/BMSCs, GelMA-T scaffolds loaded with bone mesenchymal cells; H&E, hematoxylin and eosin; NS, nasal septum; SO, safranine-O; TB, toluidine blue.

As shown in Figure 5A, after repair was allowed to proceed for 12 weeks, the structure of NS cartilage in the control group was almost absent, whereas large amounts of fibrous and connective tissue composed of aggregated macrophages filled the area between the bilateral mucosal tissue. The 10% GelMA-T scaffold in the defect site was completely lost, and the structure of the NS cartilage was significantly reduced and showed a larger structural defect. Moreover, proteoglycans (red) and the chondrogenic matrix (blue) began to degrade gradually along the border of the NS cartilage (Figures 5B,C), indicating that catabolism was increased. In contrast, the structure of the NS cartilage treated with the 10% GelMA-T/BMSCs scaffold tended to be gradually reconstructed. Proteoglycans (red) filled the area between the bilateral mucosal tissues, revealing increased anabolism of the extracellular matrix of chondrocytes. Neocartilage formation (areas marked by the dashed line in Figure 5B) was also observed, which was commonly surrounded by cellular infiltrates and was morphologically differentiated from the mature cartilage by an island-like structure and low *Col II* expression (Kaiser et al., 2006; Lena et al., 2022). In addition, the cellular distribution of NS cartilage treated with the 10% GelMA-T/BMSCs scaffold was similar to that in the sham group (Figure 5A), with round mature cells restricted to the middle and flat immature cells to the periphery (Baddam et al., 2021).

**FIGURE 6:**
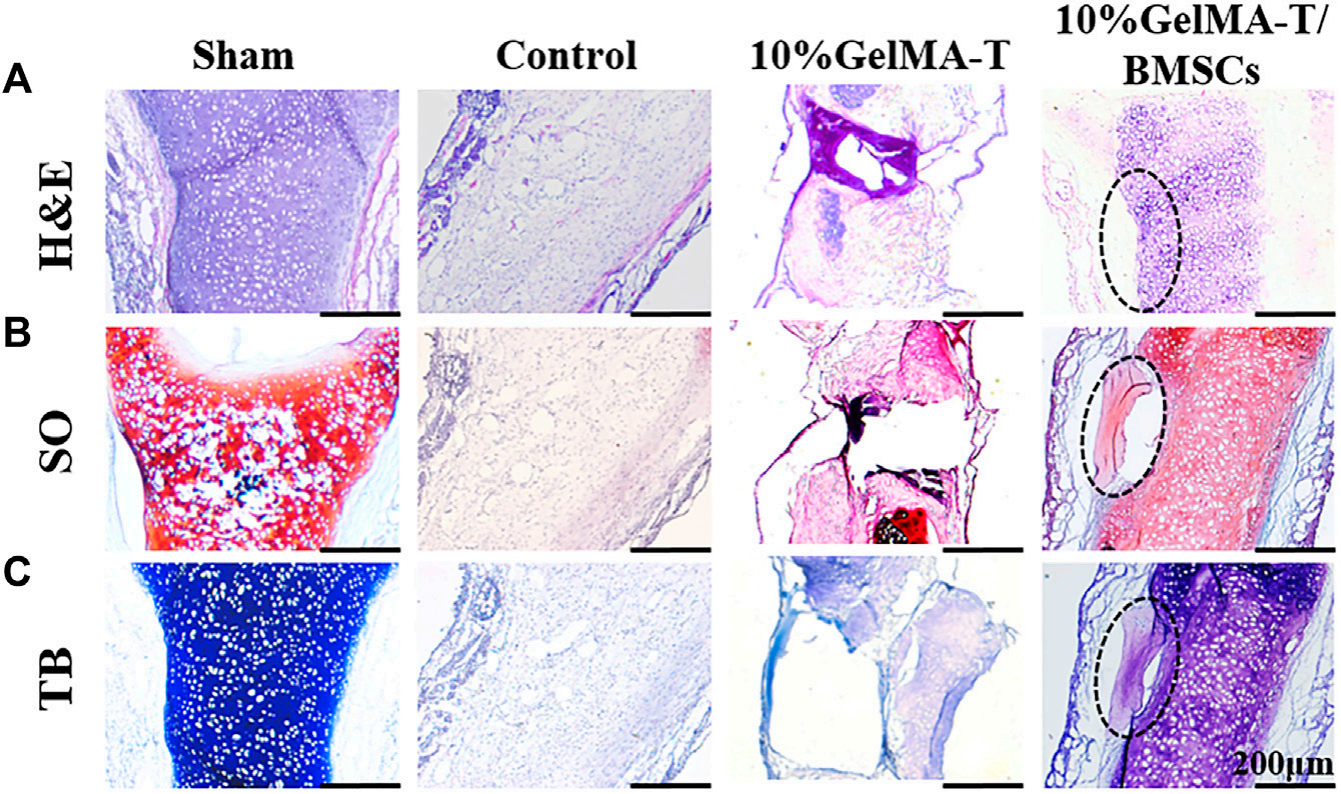
Representative image of rabbit NS cartilage repaired using 10% GelMA-T and 10% GelMA-T/BMSCs scaffolds at 24 weeks. **(A)** H&E, **(B)** SO, and **(C)** TB staining of NS cartilage in different groups. Scale bar: 200 μm. Abbreviations: GelMA-T, gelatin methacrylate-based scaffolds loaded with transforming growth factor-β1; GelMA-T/BMSCs, GelMA-T scaffolds loaded with bone mesenchymal cells; H&E, hematoxylin and eosin; NS, nasal septum; SO, safranine-O; TB, toluidine blue.

As shown in Figure 6, after repair was allowed to proceed for 24 weeks, the repair outcomes of the different groups showed clear disparities. The structure of the NS cartilage in the control group showed little change compared to that at 12 weeks, indicating that the NS cartilage was structurally replaced by fibrous and connective tissue from the bilateral mucosa. NS cartilage treated with the 10% GelMA-T scaffold exhibited a larger defect than that at 12 weeks, and the other side of the defect in the NS cartilage had thinner cartilage (Figure 6A). This result indicates that the defective NS cartilage developed in the direction of the perforation injury. In contrast, NS cartilage treated with the 10% GelMA-T/BMSCs scaffold showed a significantly decreased defect area (circle drawn in a black dashed line) and an intact and dense NS cartilage structure, similar to that in the sham group. In addition, mature chondrocytes in the sham group were round, vacuous, and pale. In contrast, neonate chondrocytes in cartilage treated with the 10% GelMA-T/BMSCs that were mainly distributed along the defect had larger and more obvious nuclei, suggesting that mitosis was occurring, along with active production of matrix (Kaiser et al., 2006). Moreover, proteoglycans (red) and the chondrogenic matrix (blue) gradually formed in the residual defect area (circle drawn by a black dashed line) (Figures 6B,C).

The mechanism of repair of the NS cartilage defect could be attributed to a synergistic effect of BMSCs and TGF‐β1. In the early phase, we observed some laden BMSCs in the residual porous structure (Figure 4, arrow), which demonstrated the successful delivery of exogenous BMSCs using the tissue engineering strategy, and solved the problem of lack of endogenous stem cells during cartilage regeneration. Additionally, Grimaud et al. (2002) proposed a positive role for TGF-β1 in promoting chondrocyte anabolism. Similarly, in our study, BMSCs in the porous structure showed high expression of chondrogenic matrix, likely derived from the beneficial effects of TGF‐β1 in promoting anabolism of BMSCs and chondrocytes. The biological mechanism of these effects will be further investigated in our future studies.

## Conclusion

1) Through this research, we developed a new kind of photo-crosslinked hydrogel scaffold loaded with TGF-β1 and BMSCs for repairing NS cartilage defects.
2) The hydrogel scaffolds had appropriate stiffness, high porosity, and the capability for sustained release of TGF-β1, which provided advantageous conditions for cell adhesion and induced chondrogenic differentiation of rabbit-derived BMSCs *in vitro.*
3) With BMSC loading, the tissue-engineered hydrogel scaffolds supported the filling and repair of defects in NS cartilage.
4) This research provides an alternative method to induce the regeneration and repair of NS cartilage.

## Data Availability

The original contributions presented in the study are included in the article/Supplementary Material; further inquiries can be directed to the corresponding authors.
