# Supplementary material for: A TGF-loading hydrogel scaffold capable of promoting chondrogenic differentiation for repairing rabbit nasal septum cartilage defect
Source: Front Bioeng Biotechnol. 2022 Nov 18;10:1057904. doi: 10.3389/fbioe.2022.1057904 (PMC9717702; doi:10.3389/fbioe.2022.1057904)
Supplement: Supplementary file 1 [file DataSheet1.docx]

Supplementary Material

A TGF-loading hydrogel scaffold capable of promoting chondrogenic differentiation for repairing rabbit nasal septum cartilage defect

Dan Zhang^1, †^, Ying Su^1, 3, †^, Peng Sun^1, †^, Xingzhi Liu^2^, Lin Zhang^2^, Xuwei Ling^2^, Yuhui Fan^1^, KangWu^2, *^, Qin Shi^2, *^, Jisheng Liu^1, *^

^1^Department of Otolaryngology, The First Afﬁliated Hospital of Soochow University, Soochow University, Suzhou, Jiangsu, China

^2^Department of Orthopaedics, The First Afﬁliated Hospital of Soochow University, Orthopaedic Institute, Soochow University, Suzhou, Jiangsu, China

^3^Department of Otolaryngology, The Afﬁliated Suzhou Hospital of Nanjing Medical University, Suzhou Municipal Hospital, Gusu School, Nanjing Medical University, Suzhou, China.

† **These authors contributed equally to this work.**

**^*^ Corresponding authors:**
E-mail: w151001119@163.com (**K. Wu**), shiqin@suda.edu.cn (**Q. Shi**), sdfyyljs@sina.com (**J. Liu**)

# Supplementary Table

**TABLE S1.** Primers used for qRT-PCR.

| **Gene** | **Sequences** | |
| --- | --- | --- |
| *GAPDH* | | Forward (5' - 3'): GTCGGAGTGAACGGATTTG |
|  |  | Reverse (5' - 3'): GTAGACCATGTAGTGGAGGT |
| *Col II* | | Forward (5' - 3'): TTCTCCTTTCTGCCCCTTTGGT |
|  |  | Reverse (5' - 3'): TCTGTGAAG ACACCA AGGACTG |
| *Sox9* | | Forward (5' - 3'): CTCCGACACCGAGAATACA |
|  |  | Reverse (5' - 3'): CCTCTTCGCTCTCCTTCTT |
| *ACAN* | | Forward (5' - 3'): CAGCCGGACAACTTCTTT |
|  |  | Reverse (5' - 3'): GTGAAGGGTAGGTGGTAATTG |

# Supplementary Figures


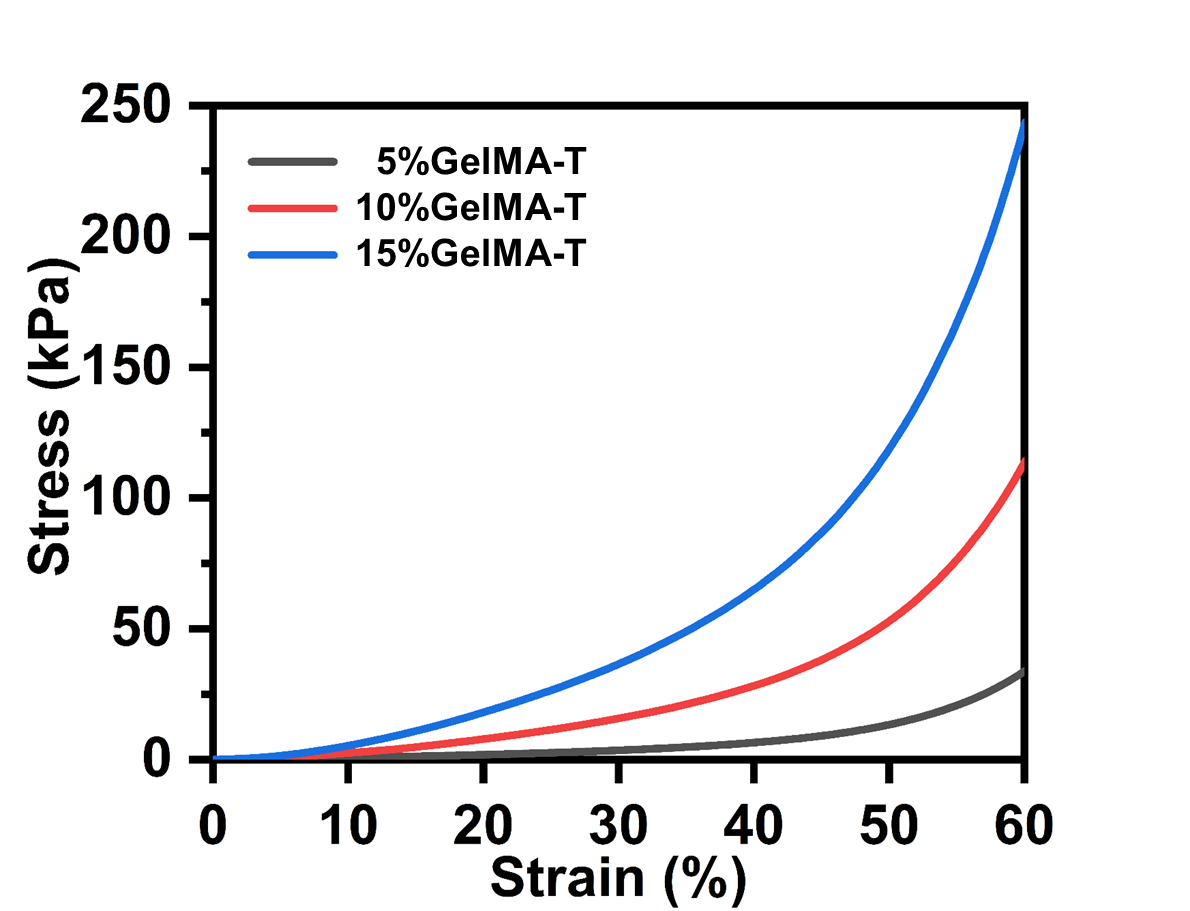


**FIGURE S1.** The compressive stress-strain curves of 5%, 10% and 15% GelMA-T scaffolds.

**FIGURE S2.** The swelling ratio (SR) of three GelMA-T scaffolds in deionized water for 24 h.

**
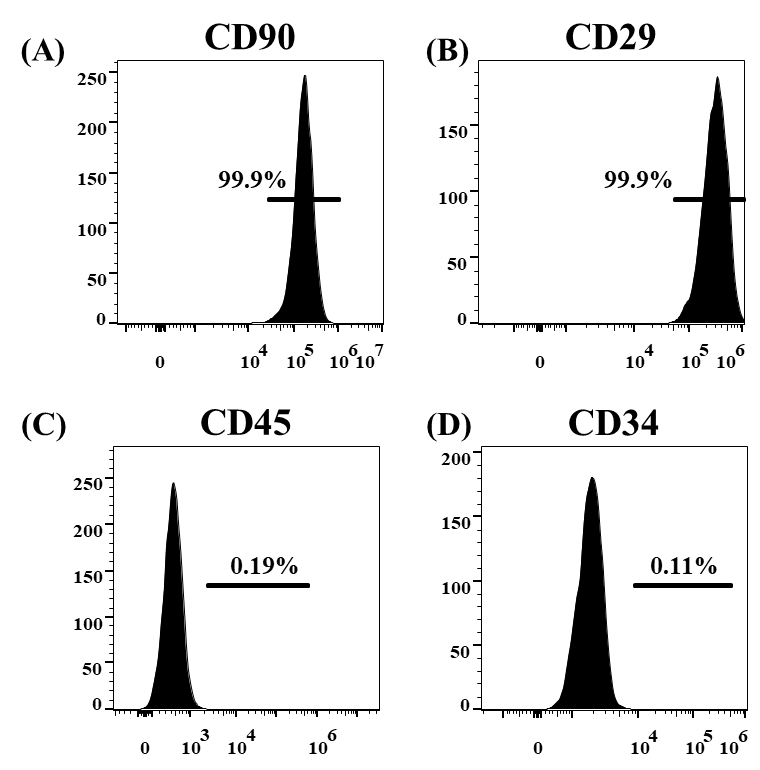
**

**FIGURE S3.** (A)-(D) The immunophenotyping results (CD90, CD29, CD45 and CD34) of bone marrow mesenchymal stem cells (BMSCs).


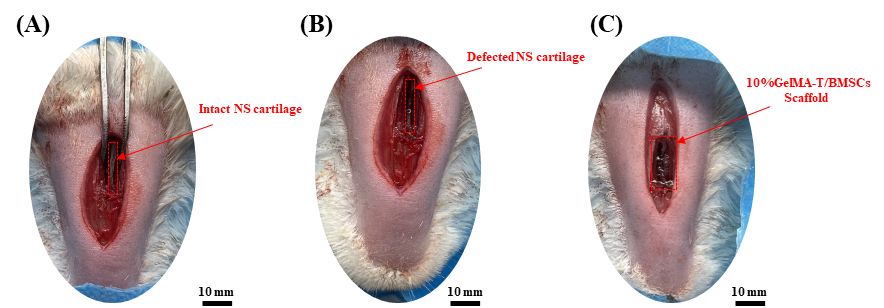


**FIGURE S4.** (A) Intact NS cartilage. (B) Defected NS cartilage. (C) Defected NS cartilage filled with 10% GelMA-T/BMSCs scaffold.
